# Analysis of the heat shock response in mouse liver reveals transcriptional dependence on the nuclear receptor peroxisome proliferator-activated receptor α (PPARα)

**DOI:** 10.1186/1471-2164-11-16

**Published:** 2010-01-07

**Authors:** Beena Vallanat, Steven P Anderson, Holly M Brown-Borg, Hongzu Ren, Sander Kersten, Sudhakar Jonnalagadda, Rajagopalan Srinivasan, J Christopher Corton

**Affiliations:** 1 NHEERL Toxicogenomics Core, US EPA, Research Triangle Park, NC 27711, USA; 2 Safety Assessment, Merial, Ltd., Duluth, GA 30096, USA; 3 Department of Pharmacology, Physiology and Therapeutics, University of North Dakota, School of Medicine, 501 N. Columbia Road, Grand Forks, ND 58203-2817, USA; 4 Nutrition, Metabolism and Genomics Group, Wageningen University and Nutrigenomics Consortium, TI Food and Nutrition, Wageningen, the Netherlands; 5 Institute of Chemical and Engineering Sciences, A*STAR (Agency for Science, Technology and Research), 1 Pesek Road, Jurong Island, Singapore; 6 Department of Chemical and Biomolecular Engineering, National University of Singapore, 10 Kent Ridge Crescent, Singapore

## Abstract

**Background:**

The nuclear receptor peroxisome proliferator-activated receptor alpha (PPARα) regulates responses to chemical or physical stress in part by altering expression of genes involved in proteome maintenance. Many of these genes are also transcriptionally regulated by heat shock (HS) through activation by HS factor-1 (HSF1). We hypothesized that there are interactions on a genetic level between PPARα and the HS response mediated by HSF1.

**Results:**

Wild-type and PPARα-null mice were exposed to HS, the PPARα agonist WY-14,643 (WY), or both; gene and protein expression was examined in the livers of the mice 4 or 24 hrs after HS. Gene expression profiling identified a number of *Hsp *family members that were altered similarly in both mouse strains. However, most of the targets of HS did not overlap between strains. A subset of genes was shown by microarray and RT-PCR to be regulated by HS in a PPARα-dependent manner. HS also down-regulated a large set of mitochondrial genes specifically in PPARα-null mice that are known targets of PPARγ co-activator-1 (PGC-1) family members. Pretreatment of PPARα-null mice with WY increased expression of PGC-1β and target genes and prevented the down-regulation of the mitochondrial genes by HS. A comparison of HS genes regulated in our dataset with those identified in wild-type and HSF1-null mouse embryonic fibroblasts indicated that although many HS genes are regulated independently of both PPARα and HSF1, a number require both factors for HS responsiveness.

**Conclusions:**

These findings demonstrate that the PPARα genotype has a dramatic effect on the transcriptional targets of HS and support an expanded role for PPARα in the regulation of proteome maintenance genes after exposure to diverse forms of environmental stress including HS.

## Background

Physiological and chemical stresses produce disease states in which proteins are damaged or misfolded in part through increases in oxidative stress. Many endogenous pathways are engaged in restoring cellular homeostasis, including stabilization of unfolded proteins to prevent aggregation and removing damaged or excess proteins through proteolysis. Stabilization of unfolded proteins is performed by molecular chaperones that assist in the folding of nascent polypeptides. Many genes encoding for chaperones exhibit increased expression after exposure to a wide variety of stimuli including chemical exposure or increased temperatures and are thus called heat shock (HS) protein (*Hsp*) genes [1-3]. These proteins play key roles in a number of human diseases [4]. Expression of some *Hsp *is essential for cellular survival under physical or chemical exposure conditions that increase oxidative stress [5,6]. Regulation of the *Hsp *genes by heat or chemical-induced oxidative stress is controlled in part by HS factor 1 (HSF1), activated under conditions in which the level of unfolded proteins increase [1-3]. Microarray studies of mouse embryonic fibroblasts from wild-type and HSF1-null mice or a human cervical carcinoma cell line have shown that HSF1 controls only a subset of the genes altered by HS [7,8] indicating that other inducible pathways play roles in regulating the *Hsp *genes.

The nuclear receptor peroxisome proliferator-activated receptor α (PPARα) is one of three PPAR subtypes that regulate lipid and glucose homeostasis, tissue growth and inflammation after exposure to a large class of structurally heterogeneous industrial and pharmaceutical chemicals called peroxisome proliferator chemicals (PPC) [9,10]. The PPARα subtype plays a key role in mediating the effects of hypolipidemic and xenobiotic PPC in liver, kidney, heart and skin. Exposure to PPC leads to regulation of a large number of genes including up-regulation of those involved in lipid homeostasis and down-regulation of inflammatory genes that are generally abolished in PPC-exposed PPARα-null mice [11-13].

There is compelling evidence that PPARα protects tissues from chemical-induced oxidative stress (reviewed in [14]). Prior exposure of rats and mice to PPC protects the liver from damage by cytotoxic agents that induce oxidative stress [15]. The hypolipidemic drug and PPC, clofibrate, protects the liver from damage by the cytotoxicant acetaminophen in wild-type but not PPARα-null mice [16]. Compared to wild-type mice, untreated PPARα-null mice or primary hepatocytes isolated from PPARα-null mice were more sensitive to carbon tetrachloride-, paraquat- or cadmium-induced toxicity [12]. The beneficial effects of caloric restriction in protecting the liver from cytotoxicant-induced liver injury were shown to depend on PPARα [17]. Additional studies have shown that PPARα plays a positive role in recovering from partial hepatectomy [18]. In the kidney, PPARα-null mice were more sensitive to damage after ischemia-reperfusion injury, and prior exposure of wild-type mice to PPC reduces the injury [19,20]. Our previous microarray studies identified an overlap in the genes regulated by the PPARα agonist WY-14,643 (WY) and those regulated by HS through HSF1 [12]. The proteome maintenance genes included those involved in protein folding (e.g., *Hsp *genes) as well as ubiquitin-dependent and -independent proteolytic processing through the proteosome (e.g., *Psm *genes). Altered regulation of these genes by PPC could help to explain why PPC exposure through PPARα helps to protect tissues from environmental stressors.

In the present study, we hypothesized that there were interactions on a genetic level between PPARα and the HS response mediated by HSF1. We dissected the contribution of PPARα by examining gene expression changes in the livers of wild-type and PPARα-null mice after HS. We found that a number of genes regulated by HS were dependent on PPARα for regulation. Furthermore, we showed that PPARα-null mice were more sensitive to the transcriptional effects of HS and exhibited a remarkably different transcriptional response compared to wild-type mice. Our findings suggest PPARα is a major regulator of stress responses in the liver.

## Methods

### Animals, exposure to heat and WY

All animal studies were carried out at CIIT Centers for Health Research, Research Triangle Park, NC. Studies utilized wild-type and PPARα-null male mice 9-12 weeks of age on a mixed SV129/C57BL/6 background. These mice have been described previously [21]. The mice were originally obtained from Dr. Frank Gonzalez, NCI, National Institutes of Health to establish a breeding colony at CIIT. Control and treated mice were provided with NIH-07 rodent chow (Zeigler Brothers, Gardeners, PA) and deionized, filtered water *ad libitum*. Lighting was on a 12-hr light/dark cycle. Mice (n = 5-6 per group) were fed a control diet or a diet containing the PPARα agonist WY-14,643 (ChemSyn Science, Lenexa, KS) (WY) in the diet (500 ppm) for 1 week followed by a 40-min heat stress at 42°C or held at room temperature in wire rack cages. Mice were sacrificed 4 hrs, 24 hrs and 3 days after the heat stress.

Heat in the exposure chamber was generated by a combination of the building reheat system, an in-line air heater and two small surface heaters. The in-line heater (Riley Equipment Company, Inc., Houston, TX) was set to approximately 300°F. The heated air traveled through a 2 foot by 2 inch inlet tube constructed of PVC, stainless steel and Teflon into the bottom of the H1000 inhalation chamber. A small diverter was placed in the bottom of the H1000 inhalation chamber to prevent a chimney effect in the chamber and increasing the efficiency of the heat distribution. The stainless steel animal cage unit was placed in the center of the H1000 inhalation chamber and a feces/urine catch pan was placed in the chamber. A small (1500 watt) heater was placed on either side of the H1000 inhalation chamber approximately 4 inches from the surface. These heaters heated the stainless steel sides and heated the surface enough to allow the internal temperature of the chamber to reach approximately 42°C. The temperature was monitored in three locations of the H1000 inhalation chamber: front door above the animal cage unit, back door below the animal cage unit and in the center using temperature probes (Rotronic Hygrometer Series 1200, Rotronic Instrument Corp., Huntington, NY).

Portions of the livers were rapidly snap-frozen in liquid nitrogen and stored at -70°C until analysis. Where appropriate, slices of liver were fixed in 10% neutral buffered formalin for 48 h, transferred to 70% ethanol, and embedded in paraffin; 5-μm sections were cut and mounted on slides and stained with H&E. H&E-stained liver sections were examined by light microscopy. Clinical chemistry markers in the serum from animals were measured by Antech Diagnostics, Charlotte, NC. All animal studies were conducted under federal guidelines for the use and care of laboratory animals and were approved by Institutional Animal Care and Use Committees.

### RNA Isolation and Analysis of Gene Expression

Liver RNA was isolated using a modified guanidium isothiocyanate method (TRIzol^®^; Invitrogen) and was further purified using silica membrane spin columns (RNeasy^®^; Qiagen, Valencia, CA). RNA integrity was assessed by the RNA 6000 LabChip^® ^kit using a 2100 Bioanalyzer (Agilent Technologies, Palo Alto, CA). Global gene expression changes were examined using the Affymetrix platform. Gene expression changes were assessed in the livers from three mice in each of the 8 treatment groups in wild-type and PPARα-null mice using a total of 24 chips. Gene expression in each animal was assayed on a separate chip. Biotin-labeled cRNA was produced from 15 μg total RNA using an Affymetrix "one-way" labeling kit. Total cRNA was quantified using a Nano-Drop ND-1000 spectrophotometer (NanoDrop Technologies, Wilmington, DE) and evaluated for quality after fragmentation on a 2100 Bioanalyzer. Following overnight hybridization at 45°C to Affymetrix U74Av2 GeneChips in an Affymetrix Model 640 GeneChip hybridization oven, the arrays were washed and stained using an Affymetrix 450 fluidics station as recommended by the manufacturer and scanned on an Affymetrix Model 3000 scanner. After scanning, raw data (Affymetrix .cel files) were obtained using Affymetrix GeneChip Operating Software (version 1.4). All of the Affymetrix (Santa Clara, CA) .cel files were analyzed by Bioconductor SimpleAffy to assess data quality [22].

Data (.cel files) was analyzed and statistically filtered using Rosetta Resolver^® ^version 7.1 software (Rosetta Inpharmatics, Kirkland, WA). Statistically significant genes were identified using one-way ANOVA with a false discovery rate (Benjamini-Hochberg test) of ≤ 0.05 followed by a post-hoc test (Scheffe) for significance. Significant transcripts were evaluated for relevance to canonical pathways and biological functions using Ingenuity Pathways Analysis (Ingenuity Systems, http://www.ingenuity.com). Heat maps were generated using Eisen Lab Cluster and Treeview software http://rana.lbl.gov/EisenSoftware.htm.

Gene Set Enrichment Analysis (GSEA; http://www.broad.mit.edu/gsea/) was used to evaluate whether a pre-defined set of genes showed statistically significant, concordant differences between two biological states [23]. GSEA generates a list of genes from submitted .cel files and tests whether, within that queried list of genes, there is a statistically significant enrichment or not of pre-defined groups of genes, or "gene sets". Gene sets examined through GSEA include canonical, metabolic and signaling pathways as well as groups of genes previously identified and validated to be up- or down-regulated when cells are given a particular stimulus. This type of analysis can sometimes detect more subtle changes present in the data. Expression profiles were submitted to GSEA using default settings and searched for enriched gene sets. Gene set C2 (curated gene sets) includes genes from online pathway databases, publications in PubMed, and knowledge from domain experts. Gene set C3 (motif gene sets) contains genes that share a transcription factor (TF) binding site defined in TRANSFAC^®^, a database for transcription factors and their genomic binding sites. Additional .cel files were analyzed using GSEA including those from the livers of wild-type and PPARα-null mice exposed to WY (400 uL of a 10 mg/mL solution/day) for 6 hr or 5 days [24].

A description of the HS and WY exposure microarray experiment is available through Gene Expression Omnibus (GEO) at the National Center for Biotechnology Information http://www.ncbi.nlm.nih.gov/geo/, as accession number GSE14,869.

We categorized the genes regulated by HS in wild-type or PPARα-null mice based on whether they were altered by HS in mouse embryonic fibroblasts (MEF) from wild-type or HSF1-null mice [8]. From this dataset, genes which fell into three groups were identified including 1) HSF1-dependent regulation by HS, 2) HSF1-independent regulation by HS and 3) altered regulation between wild-type and HSF1-null MEFs in the absence of HS. Genes were identified using procedures outlined in [25].

### Real Time RT-PCR Analysis

The levels of gene expression were quantified using real time RT-PCR analysis. Real-time reverse transcriptase-PCR was performed as follows. After DNase treatment, total RNA was quantified (Ribogreen^®^, Molecular Probes, Inc., Eugene, OR) and diluted with water. Fifty ng of RNA and PCR reagents were aliquoted into 96-well plates using an ABI Prism™ 6700 Automated Nucleic Acid Workstation (Applied Biosystems, Foster City, CA) and subjected to real-time quantitative PCR (TaqMan^®^, Applied Biosystems) using gene-specific primers (Additional File 1) and fluorescently labeled probes (Molecular Probes) designed by the Primer Express^® ^software (Applied Biosystems). Amplification curves were generated using the ABI Prism™ 7900 HT Sequence Detection System (Applied Biosystems). Expression relative to vehicle control animals was determined after normalizing to the ribosomal *18S *gene. There were four animals per treatment group, and each sample was analyzed in duplicate. Variability is expressed as standard error of the mean. Means and S.E. (*n *= 4) for RT-PCR data were calculated by Student's *t *test. The level of significance was set at *p *≤ 0.05.

### Western Blot Analysis

Liver lysates were prepared in 250 mM sucrose, 10 mM Tris-HCl, pH 7.4, and 1 mM EDTA with protease inhibitors (0.2 mM phenylmethylsulphonyl fluoride, 0.1% aprotinin, 1 μg/ml pepstatin, 1 μg/ml leupeptin) as previously described [26]. Fifty μg hepatocyte whole cell lysate was subjected to 12% sodium dodecyl sulfate - polyacrylamide gel electrophoresis followed by transfer to nitrocellulose membranes. Immunoblots were developed using primary antibodies against acyl-CoA oxidase (ACO) (a gift from S. Alexson, Huddinge University Hospital, Huddinge, Sweden), HS proteins (Santa Cruz Biotechnology, Santa Cruz, CA; StressGen, Victoria, B.C., Canada) or CYP4A (GenTest, Waltham, MA) and appropriate secondary antibodies conjugated with horseradish peroxidase (Santa Cruz Biotechnology) in the presence of chemiluminescent substrate ECL (Amersham, Piscataway, NJ). Blots were quantitated using Gel-Pro (MediaCybernetics, Silver Spring, MD). Most antibodies recognized only one major band of the expected size. Antibodies to TCP1η routinely gave 2 bands of ~60 kDa and ~40 kDa, both of which were elevated after HS, WY or both treatments. In this study we report the levels of ACO-B protein, the 52 kDa processed form of the full-length ACO protein (ACO-A) [27].

## Results and Discussion

### Identification of PPARα-dependent heat shock-regulated genes

**Figure 1:**
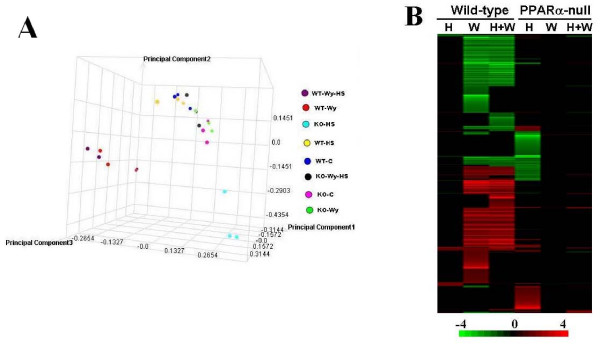
**Altered gene expression by heat shock and WY in wild-type and PPARα-null mice**. Mice were fed a control diet or a diet containing WY for 7 days. Groups of mice were subjected to a 42°C HS for 40 min or kept at room temperature. Mice were sacrificed 4 hrs after HS and hepatic mRNA levels were assessed in the livers. A. Principle component analysis. B. Heat map of altered gene expression. Genes were subjected to one-dimensional hierarchical clustering. Red, up-regulation; green, down-regulation; black, no change. The intensity scale indicates fold-change due to chemical exposure relative to controls. Abbreviations: H, heat shock; W, WY-14,643.

We previously identified chaperone genes regulated by WY through PPARα that are also targets of HS through HSF1 [12]. We set out to characterize the HS response in the mouse liver and to determine if there were interactions between HS and PPARα. Wild-type and PPARα-null mice were exposed to either a WY diet or a control diet for 7 days and then groups of mice were challenged with HS (42°C) for 40 minutes or kept at room temperature. Transcript profiles were determined in the livers of mice sacrificed 4 hours after HS, as this time was shown to maximally induce the HS responsive genes [28]. Principle component analysis (PCA) of the 24 chips in the study revealed the greatest differences were caused by WY or WY+HS in wild-type mice or by HS in PPARα-null mice (Figure 1A). More subtle differences were caused by HS in wild-type mice and by WY+HS in PPARα-null mice. Consistent with the PCA results, HS, WY alone or WY+HS treatments in wild-type mice altered 107, 1714 or 1418 genes, respectively (Figure 1B). Exposure to HS in wild-type mice altered a number of well-characterized targets of HSF1 (described below). Almost half of the HS regulated genes were also regulated by WY in the same direction. (The complete list of genes is found in Additional File 2.)

The gene expression pattern after treatment was dramatically different in PPARα-null mice. HS, WY alone or WY+HS treatments in PPARα-null mice altered 774, 6, or 68 genes, respectively (Figure 1B).

**Figure 2:**
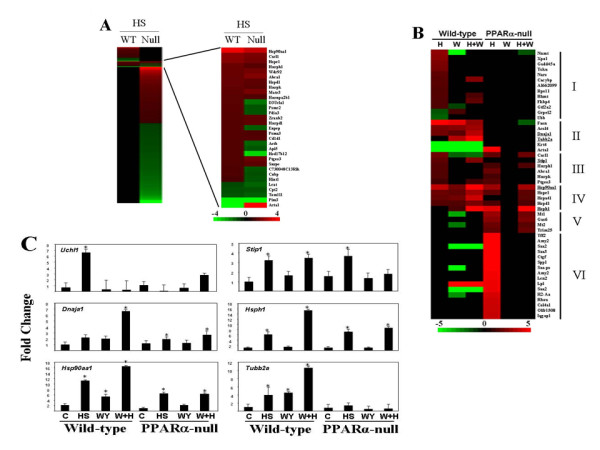
**Heat shock alters the expression of different sets of genes in wild-type and PPARα-null mice**. A. Direct comparison of HS genes in wild-type and PPARα-null mice. The relatively small number of genes which exhibited altered expression in both strains are shown in detail. B. Different classes of HS genes. The genes altered by HS were divided into six classes (I - VI) based on expression in wild-type and PPARα-null mice and comparison with WY. The genes whose expression was confirmed by TaqMan are underlined. Red, up-regulation; green, down-regulation; black, no change. The intensity scales indicates fold-change due to exposure relative to controls. C. Altered expression of genes assessed by TaqMan. There were four animals per treatment group, and each sample was analyzed in duplicate. Variability is expressed as standard error of the mean. Means and S.E. (*n *= 4) for RT-PCR data were calculated by Student's *t *test. The level of significance was set at *p *≤ 0.05.

We directly compared the genes that were regulated by HS in the two strains (Figure 2A). A small number of genes exhibited concordant expression changes in both strains including *Hsp90aa1*, *Hspe1*, and *Hspd1*. Other genes exhibited up-regulation in wild-type mice but down-regulation in PPARα-null mice. There were 76 genes that exhibited altered expression only in wild-type mice, whereas 743 genes were altered only in PPARα-null mice. For the most part, the HS genes unique to each strain lacked inclusion of Hsp family members.

The HS genes were categorized based on their expression behavior in the two strains after HS and WY exposure (Figure 2B). Genes in class I and II were altered by either HS alone (class I) or by HS and WY (class II) in a PPARα-dependent manner. The class I genes included FK506 binding protein 4 (*Fkbp4*) and GrpE-like 2, mitochondrial gene (*Grpel2*). Also included was ubiquitin carboxy-terminal hydrolase L1 (*Uchl1*) identified using less stringent microarray analysis cut-offs but confirmed by RT-PCR (Figure 2C). The class II genes included *Dnaja1 (Hsp40) *and a tubulin family member (*Tubb2a*). RT-PCR revealed concordance with array expression for *Tubb2a*; *Dnaja1 *exhibited weak induction by HS in PPARα-null mice. Genes in class III were altered only by HS and were partially, if not completely independent of PPARα, including stress-inducible phosphoprotein-1 (*Stip1*) in which increases in expression after HS in PPARα-null mice were detected by RT-PCR. Genes in class IV were induced by HS independent of PPARα and also exhibited PPARα-dependent WY induction. These genes included a number of chaperones *Hsp90aa1*, *Hspe1*, *Hspa4l*, *Hspd1 *and *Hsph1*. The expression pattern of *Hsp90aa1 *and *Hsph1 *was confirmed by RT-PCR. Class V genes were those that exhibited altered expression only in PPARα-null mice after HS but not WY and included two metallothionein genes (*Mt1, Mt2*). Class VI genes were those that exhibited altered regulation after HS in PPARα-null mice but not after HS+WY indicating suppression of HS transcriptional effects by WY. These genes included the up-regulation of serum amyloid protein family members (*Saa2, Saa3*) and down-regulation of a large number of mitochondrial genes (described below). Overall, the data is consistent with a subset of 1) HS-regulated genes (Class I and II) that are dependent on PPARα for altered expression, 2) HS-regulated genes (Class III and IV) that are independent of PPARα and 3) genes responsive to HS only in the absence of PPARα (class V and VI).

### Heat shock activates different biological responses in wild-type and PPARα-null mice

We identified sets of genes that exhibited altered regulation in the two strains using Gene Set Enrichment Analysis (GSEA). GSEA is an analysis method that evaluates the expression of biological pathways on defined gene sets, as an alternative to examining individual genes, to assist in identifying significant biological changes in microarray data sets [23]. Consistent with HS effects on proteome maintenance genes, HS in wild-type mice increased the expression of genes involved in protein synthesis (HSA03010_RIBOSOME, RIBOSOMAL_PROTEINS, TRNA_SYNTHETASES) and degradation (HSA03050_PROTEASOME, PROTEASOME_DEGRADATION, PROTEASOMEPATHWAY, PROTEASOME) (Additional File 3). There were no gene sets that exhibited significant overlap with the down-regulated HS wild-type genes.

HS in PPARα-null mice altered the expression of genes in a different set of biological categories (Additional File 4) including the up-regulation of genes associated with mouse liver tumors (LEE_DENA_UP, LEE_MYC_E2F1_UP, LEE_E2F1_UP, LEE_MYC_TGFA_UP, LEE_ACOX1_UP, LEE_CIP_UP, LEE_MYC_TGFA_DN), muscle contraction (STRIATED_MUSCLE_CONTRACTION) and the aging kidney (AGEING_KIDNEY_UP).

There were a large number of genes down-regulated by HS in PPARα-null mice associated with mitochondrial biogenesis and function regulated by the PPARγ Co-activator 1 (PGC-1) family which includes PGC-1α, PGC-1β and PRC (MOOTHA_VOXPHOS, PGC, HUMAN_MITODB_6_2002, MITOCHONDRIA) (Additional File 5). There was also down-regulation of genes involved in mouse and human liver tumors (LEE_MYC_E2F1_DN, LEE_DENA_DN, LEE_MYC_TGFA_DN, LEE_CIP_DN, HCC_SURVIVAL_GOOD_VS_POOR_UP) and down-regulation of genes associated with amino acid and fatty acid metabolism (VALINE_LEUCINE_AND_ISOLEUCINE_DEGRADATION, HSA00280_VALINE_LEUCINE_AND_ISOLEUCINE_DEGRADATION, HSA00380_TRYPTOPHAN_METABOLISM, HSA00071_FATTY_ACID_METABOLISM). The fatty acid metabolism genes included those that are regulated by PPARα upon exposure to PPC (discussed below).

GSEA was also used to identify candidate transcription factors responsible for altered regulation of the HS genes. A number of stress-inducible transcription factors are likely involved in the altered expression of HS genes in wild-type mice. Consistent with well-characterized mechanisms of HS gene regulation, GSEA twice identified HSF1 as a candidate transcription factor of the HS genes (Additional File 6). Two sets of genes positively regulated by HS overlapped with those regulated by X-box binding protein (XBP1). XBP1 is a key component of the endoplasmic reticulum (ER) stress response [29]. Accumulation of unfolded proteins in the ER activates the unfolded protein response (UPR), resulting in transcriptional induction of ER chaperones and proteases in part through activation of XBP1. A number of genes associated with XBP1 were either up- (HOXC4, STAT3, PAK1IP1, SGK, VDP, SEC61A1, SFRS2, UBQLN1, MORF4L2) or down-regulated (COL1A2, ATBF1, POLG, GRIA3) in wild-type mice. HS genes linked to up-regulation of nuclear respiratory factor 1 (NRF1) were also identified (data not shown). NRF1 is activated by increased levels of heme and regulates a large number of genes involved in mitochondrial biogenesis and oxidative stress that overlap with those regulated by PGC-1α or PGC-1β[30,31].

In contrast, the genes regulated by HS in PPARα-null mice overlapped with serum response factor (SRF) (one set significantly and three approaching significance) (Additional File 7). SRF is a ubiquitously expressed transcription factor that plays a role in regulating early response genes to various stimuli including healing after tissue injury [32]. SRF plays a prominent role in regulating the expression of heart muscle genes [33] and may explain increases in muscle contraction genes (ACTA1, ACTA2, DES, MYL1, MYL2, MYL3, MYL9, MYOM1, TNNC2, TNNT1, TNNT3, TPM2, TPM4) after HS. Two gene sets that approached significance were regulated by HSF1. In summary, these results indicate that the PPARα genotype has a dramatic effect on the biological pathways that are affected by HS.

### PPARα-independent changes in triglyceride levels and fatty acid oxidation protein expression by heat shock

**Figure 3:**
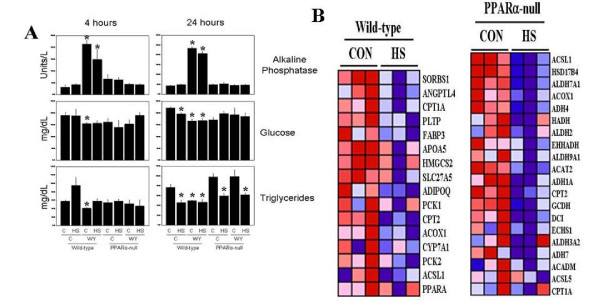
**PPARα-independent changes in triglyceride levels and fatty acid metabolism genes by heat shock**. A. Serum levels of alkaline phosphatase, glucose and triglycerides 4 and 24 hrs after HS. B. Decreases in fatty acid metabolism genes by HS in wild-type and PPARα-null mice. Genes called as significantly altered between control and HS groups were extracted from GSEA gene sets HSA03320_PPAR_SIGNALING_PATHWAY (wild-type mice) and HSA00071_FATTY_ACID_METABOLISM (PPARα-null mice).

We examined the levels of a number of clinical chemistry markers including triglycerides in the blood from mice 4 or 24 hrs after HS. The levels of alkaline phosphatase were increased in all WY-treated wild-type mice but not PPARα-null mice (Figure 3A), paralleling the increases in gene expression for alkaline phosphatase, liver/bone/kidney (*Alpl*) after exposure to WY and other PPC (data not shown). Consistent with PPARα agonists playing a role in glucose homeostasis [34,35], glucose levels were lower in wild-type but not PPARα-null WY-treated mice at 4 and 24 hrs. HS also lowered glucose levels in wild-type but not PPARα-null mice at 24 hrs. HS did not induce overt liver damage as measured by ALT levels; minor increases were observed by HS+WY treatment in wild-type but not PPARα-null mice at 4 and 24 hrs that approached significance (data not shown). These results were consistent with a lack of pathological findings in the liver. In wild-type mice triglyceride levels were increased by HS and decreased by WY at 4 hrs with no change by both treatments. At 24 hrs, triglyceride levels were decreased by HS, WY or HS+WY in wild-type mice and by HS or HS+WY in PPARα-null mice. Despite uniform increases in cholesterol biosynthetic genes after WY exposure that were reversed by WY+HS treatment (Additional File 8 and 9), no changes in circulating cholesterol were noted between control and treated groups for each strain (data not shown).

**Figure 4:**
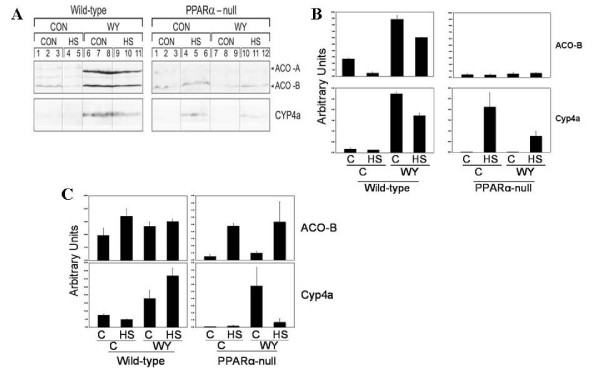
**Expression of Acox1 and Cyp4a proteins after WY and heat shock in livers and kidneys**. A. Expression of ACO and Cyp4a in livers of wild-type or PPARα-null mice on control or WY diet, 24 hrs after HS or mock HS. Expression was assessed by Western blot using primary antibodies against the indicated proteins. B. Quantitation of the western blots in C. C. Altered protein expression in the kidneys of wild-type or PPARα-null mice on control or WY diet, 24 hrs after HS or mock HS. Expression was assessed by Western blot using primary antibodies against the indicated proteins.

Triglyceride levels are controlled in part by enzymes involved in fatty acid oxidation. Expression of fatty acid metabolism genes/proteins and triglyceride levels are reciprocally related. HS decreased the transcript levels of a number of genes involved in fatty acid transport and metabolism in both mouse strains including the *Acox1 *gene (Figure 3B). In wild-type mice we observed the expected increases in enzymes involved in fatty acid oxidation including Aco and Cyp4a after exposure to WY in wild-type but not PPARα-null mice 24 hrs after HS in liver (Figure 4A, B) and kidney (Figure 4C). HS had complex effects on expression of Aco and Cyp4a. In the liver HS decreased the expression of ACO-B protein in wild-type mice at 24 hrs. In the kidney but not the liver, ACO-B protein was increased by HS in PPARα-null mice. In the liver but not the kidney, Cyp4a protein was induced by HS only in PPARα-null mice. HS-inducible gene expression of *Cyp4a10/14 *genes was not observed by microarray indicating post-transcriptional regulation of expression.

These findings indicate that HS elicits complex changes in fatty acid metabolism gene and protein expression leading to alterations in triglyceride levels, some of which are independent of PPARα. The increases in triglyceride levels in wild-type mice by HS at 4 hrs may be attributed in part to decreases in fatty acid oxidation genes; the decreases in triglyceride levels in PPARα-null mice are correlated with increases in Cyp4a and ACO-B in the liver or kidney. The decreases in triglyceride levels in wild-type mice 24 hrs after HS cannot be explained by the decreases in ACO and Cyp4a in liver and kidney. Thus, additional mechanisms that control triglyceride levels are likely at play including effects on mitochondrial fatty acid oxidation and require further study. In summary, 24 hrs after HS the decreases in triglyceride levels were PPARα-independent whereas decreases in glucose levels were PPARα-dependent.

### Chaperone protein expression after heat shock and WY treatment

**Figure 5:**
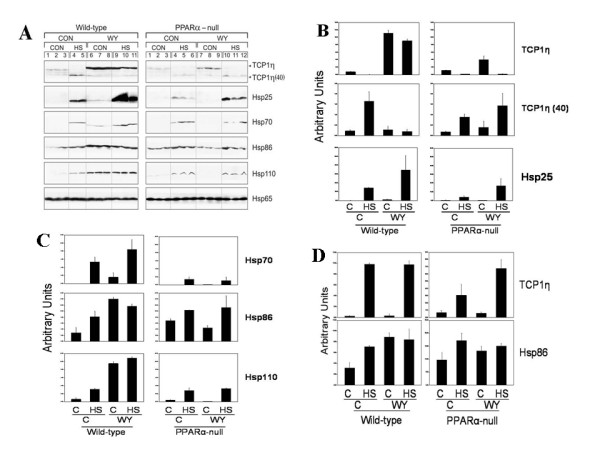
**Expression of chaperone proteins after heat shock or PPC treatment**. A. Expression of Hsp in the livers of mice. Proteins were extracted from the livers of wild-type or PPARα-null mice on control or WY diet, 24 hrs after HS or mock HS. Expression was assessed by Western blot using primary antibodies against the indicated proteins. B and C. Quantitation of the western blots in A. D. Altered protein expression in the kidneys of wild-type and PPARα-null mice exposed to HS or WY. Protein expression was assessed in the kidneys of the mice described in A.

We examined protein expression of Hsp family members in livers and kidneys of the mice 24 hrs after HS. Given the transcriptional increases in chaperonin-containing T-complex 1 (Tcp-1) family members *Cct3, Cct4, Cct7 *and *Cct8 *after exposure to WY in wild-type mice (Additional File 2), we also examined the expression of Tcp1η protein. Western blots consistently detected two bands of ~60 kDa (full-length Tcp1η) and an immunoreactive fragment of ~40 kDa (Tcp1η(40)). Increases in Tcp1η(40), Hsp25, Hsp70, Hsp86, Hsp110 but not Hsp65 were observed in wild-type mice after HS; the increases in all of the proteins were PPARα-independent, as increases were also observed in HS-treated PPARα-null mice (Figure 5A, B, C). In contrast, TCP1η exhibited decreased expression after HS in wild-type and PPARα-null mice. WY exposure led to increased expression in Tcp1η, Hsp70, Hsp86, and Hsp110 in wild-type mice that was partially or completely eliminated in PPARα-null mice. The increases in protein expression after HS and WY were generally consistent with the microarray and RT-PCR data for *Hspa1a *and *Hspa1b *(Hsp70), *Hsp90aa1 *(Hsp86/90), *Hsph1 *(Hsp105/110) and *Cct7 *(TCP1η). No transcriptional changes by microarray were detected for *Hspb1 *(Hsp25), but RT-PCR showed increases after a 7 day WY exposure in a previous study [12].

We assessed protein expression in the kidneys from the animals described in this study. Tcp1η, although not affected by HS in liver was induced up to 8-fold by HS in kidneys from both strains, indicating tissue-specific induction by HS (Figure 5D). Hsp86 but not Hsp25, Hsp70, and Hsp110 was induced by HS in the kidney (Figure 5D and data not shown). After WY exposure Hsp86, but not Tcp1η, Hsp25, Hsp70, or Hsp110 was weakly induced in the kidneys of wild-type, but not PPARα-null mice.

Most of the Hsp genes/proteins that were induced by WY (Hsp25, Hsp70, Hsp86, Hsp110, TCP1) carry out protein folding in the cytoplasm in a coordinated manner. For example, Hsp70 and Hsp110 cooperate to sequentially fold proteins in the cytoplasm [36]. In contrast, we did not observe any mitochondrial chaperones such as mtHsp70 altered on the transcriptional level; a number of chaperones which operate in the endoplasmic reticulum including BiP/Grp78 [37] and Grp94 (our study) were down-regulated. An earlier report showed that the Grp94 gene and protein were increased after exposure to WY or nafenopin [38]. Overall, our studies indicate that WY coordinately regulates a number of chaperones which operate in the cytoplasm.

What might the increased levels of chaperone proteins be doing in the liver after WY exposure? Increased levels of chaperones might allow tight control of the inducibility of PPARα. Many nuclear receptors interact with chaperone proteins including the ones induced by WY in our studies [39]. PPARα interacts with Hsp72 [40] and is inhibited by Hsp90 [41]. Thus, induction of Hsp90 and other family members may dampen the PPARα transcriptional response. Additionally, Hsp induction may help support the increases in protein synthesis associated with hepatocyte replication after PPC exposure. In addition, increased expression of Hsp family members has been associated with protection from apoptosis [42,43] and PPC, at least under acute exposure conditions decrease basal levels of apoptosis [10]. PPC exposure leads to dramatic increases in peroxisome size and number and increased expression of TCP1 subunits may be important for proper protein insertion into the peroxisomal membrane [44].

It is generally appreciated that increased expression of Hsps by mild HS can protect from subsequent challenges by more severe treatments [45]. Our microarray and protein expression analysis showed that WY can induce the gene and protein expression of a number of chaperones. These same chaperones including Hsp25/27 [46,47], Hsp70 [45,48], Hsp86/90 [49-51] and Hsp105/110 [36,52,53] when over-expressed can protect cells from various stressors. Our results identify a number of potential candidate genes/proteins that could protect cells from environmental stressors by pretreatment with PPARα agonists.

### Global decreases in mitochondrial gene expression in PPARα-null mice after heat shock are prevented by WY pretreatment

**Figure 6:**
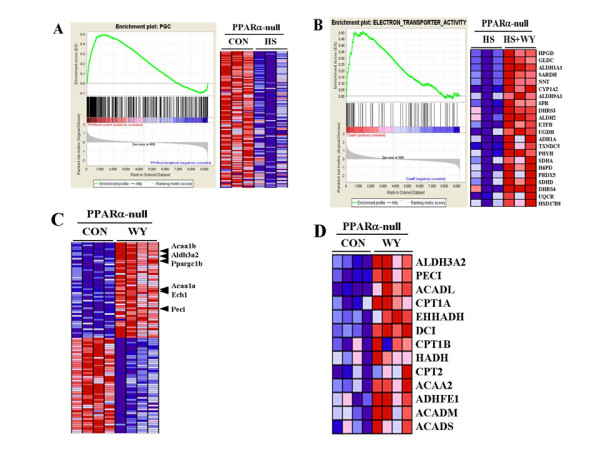
**Global decreases in mitochondrial gene expression in PPARα-null mice after heat shock are prevented by WY pretreatment**. A. Down-regulation of genes regulated by the co-activator PGC-1. Gene Set Enrichment Analysis (GSEA) was used to identify gene sets that exhibited significant overlaps with those gene differences between control and HS in PPARα-null mice. Left, enrichment plot for genes regulated by PGC-1. Black bars illustrate the position of probe sets belonging to the PGC-1 gene set in the context of all probes on the U74Av2 array. The running enrichment score (RES) plotted as a function of the position within the ranked list of array probes is shown as a green line. The ranked list metric shown in gray illustrates the correlation between the signal to noise values of all individually ranked genes according to the class labels (experimental conditions). Right, individual expression profiles for leading edge probe sets contributing to the normalized enrichment score are shown. Signal intensities are illustrated by varying shades of red (up-regulation) and blue (down-regulation). B. Prevention of down-regulation of electron transporter gene expression by pretreatment with WY. Left, enrichment plot for genes with electron transporter activity as described in A. Right, individual expression profiles for probe sets contributing to the normalized enrichment score are shown. C. Increased expression of PGC1β and regulated genes involved in lipid homeostasis by WY in PPARα-null mice. GSEA-derived heat map of the top 100 differentially expressed probe sets enriched in the control or WY-treated groups from PPARα-null mice. Location of PGC1β (*Ppargc1b*) and regulated genes are indicated. D. Increased expression of genes involved in fatty acid metabolism after exposure to WY for 5 days in PPARα-null mice. Genes significantly altered in the GSEA set "fatty acid metabolism" are shown. These include a number that overlap with those that are involved in mitochondrial fatty acid metabolism regulated by PGC-1.

HS can induce mitochondrial swelling, loss of mitochondria and uncoupling of oxidative phosphorylation [45]. HS in PPARα-null mice led to dramatic decreases in mitochondrial biogenesis and mitochondrial oxidative phosphorylation genes regulated by PGC-1 family members (Additional File 6). Coordinated down-regulation of a curated set of genes regulated by PGC-1 was observed; out of the 310 genes in this list shared by the U74Av2 array, 123 were down-regulated (Figure 6A). Pretreatment of PPARα-null mice with WY led to resistance to gene expression changes, as far fewer genes were altered by both treatments compared to HS alone (774 genes for HS alone vs. 68 genes for HS+WY). We compared the two groups directly using GSEA and identified significant increases in electron transporter genes after HS+WY treatment compared to HS alone including those that function in the mitochondria (Figure 6B). Significant alterations in mitochondrial gene sets were not observed in a comparison between control and HS+WY groups using GSEA (data not shown). Thus, WY pretreatment of PPARα-null mice prevented decreases in mitochondrial gene expression due to HS.

To identify candidates involved in resistance to HS-induced decreases in mitochondrial gene expression, we searched for genes regulated by WY independently of PPARα. Our previous experiments indicated that WY had minimal effects in the livers of PPARα-null mice, altering only up to ~8% of the total number of genes compared to wild-type mice [54]. These results were based on transcript profiles generated using the Affymetrix gene chip U74Av2 which contains ~12,000 genes. Using full-genome arrays containing ~45,000 genes (Affymetrix MOE430_2 arrays), we examined gene expression in PPARα-null mice after exposure to WY for 6 hrs or 5 days. WY had minimal effects on gene expression after 6 hrs in PPARα-null mice (1 gene change) but at 5 days, 92 genes were significantly altered in PPARα-null mice. A number of the changes would affect intermediary metabolism and mitochondrial gene expression, the most striking of these changes being increases in PGC-1β (*Ppargc1b*) (~2-fold, p = 0.01; Figure 6C). (This gene is not included on the U74Av2 array.) Genes known to be regulated by PGC-1β included *Cpt1 *[55-57] and a number of others involved in fatty acid oxidation including acetyl-Coenzyme A acyltransferase 1B (*Acaa1b*), aldehyde dehydrogenase family 3, subfamily A2 (*Aldh3a2*), acetyl-Coenzyme A acyltransferase 1A **(***Acaa1a*), enoyl coenzyme A hydratase 1, peroxisomal (*Ech1*) and peroxisomal delta3, delta2-enoyl-Coenzyme A isomerase (*Peci*) (Figure 6D).

PGC-1β is a master controller of hepatic energy homeostasis in part through regulation of mitochondrial oxidative metabolism genes. Overproduction of PGC-1β leads to increases in mitochondrial volume density in C2C12 myotubes [58], and defects in PGC-1β expression have been associated with global decreases in mitochondrial gene expression in the skeletal muscle of diabetics [30]. PGC-1β physically interacts with transcription factors involved in mitochondrial biogenesis and lipid metabolism including Nrf-1, PPARα and PPARγ [55,59,60]. In trans-activation assays, WY activates PPARβ and PPARγ, albeit to a lesser extent than PPARα [61,62]. Increases in PGC-1β co-activator function in the presence of the weak agonist function of WY may lead to increased expression of fatty acid oxidation genes regulated by PPARβ or PPARγ similar to what we have observed with the PPC perfluorooctanoic acid in PPARα-null mice [54]. These results indicate that WY can alter the expression and activity of effectors of mitochondrial biogenesis and fatty acid oxidation, preventing HS from decreasing those gene batteries in PPARα-null mice. This protective effect of WY is reminiscent of thermotolerance induced by prior treatment with a low temperature HS [45].

### *Hsp *gene regulation by PPARα and HSF1

**Figure 7:**
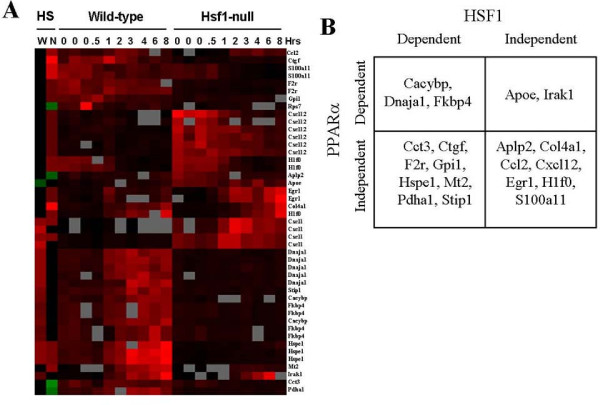
**Regulation of heat shock genes by HSF1 and PPARα**. Genes which exhibited significant changes in expression due to HS from the dataset of Trinklein et al. (2004) and from the present study were identified. A. Heat map of gene expression changes by HS in wild-type (W) and PPARα-null (N) mice compared to the Trinklein et al. (2004) dataset. In the Trinklein et al. (2004) study, mouse embryonic fibroblasts were subjected to HS followed by recovery for the indicated times in hrs. Genes were subjected to one-dimensional hierarchical clustering. Red, up-regulation; green, down-regulation; grey, no data; black, no change. B. Classification of genes based on regulation by PPARα and HSF1.

HSF1 controls expression of *Hsp *genes after HS [7,8]. We determined if there was any overlap in HS genes regulated by HSF1 and PPARα. We identified a number of classes of HSF1-dependent or -independent genes regulated by HS as well as genes that require HSF1 for basal expression (Additional File 10 and 11). We then identified genes that overlapped with those regulated by HS in wild-type and PPARα-null mice from our study. The expression of the 45 unique pairs of gene identifiers from each array is shown in Figure 7A.

The genes fell into 4 groups based on dependence on PPARα and HSF1 (Figure 7B). The largest groups were those that were PPARα-independent and were about equally split between those that were dependent or independent of HSF1. PPARα-dependent genes included those that were HSF1-dependent (*Cacybp, Dnaja1, Fkbp4*) or independent (*Apoe, Irak1*). This comparison reveals that although some genes were altered by HS independent of both PPARα and HSF1, most genes required one or both factors for HS regulation.

An important question is why does PPARα expression/activation determine in part the profile of HS genes? We hypothesize that PPARα is required for the induction of a subset of the HS genes. Class I and II genes (Figure 2B) require PPARα for HS regulation indicating that they could possess functional PPREs. Genes have been identified that possess HSEs that bind HSF1 but that were not HS-inducible. This provides evidence that other cis-acting elements or interacting factors were required for HS inducibility [8]. A linkage between HSE and PPRE in the promoters of HS genes was found as binding sites for PPAR family members were significantly increased within the 1000 bp upstream of the genes that were up- or down-regulated by HS in a human epithelial cell line [63]. Follow-up PPAR-HSF1 co-immunoprecipitation studies would reveal the nature of the interactions. Our analysis demonstrated major differences in the target genes of HS between wild-type and PPARα-null strains indicating that in the absence of PPARα, major changes in chromatin accessibility to HSF1 and other factors leads to greater sensitivity to HS alteration.

How are the HS genes regulated by PPC through PPARα? Given that *Hsp *gene expression is controlled in part by heat shock factor 1 (HSF1), one possibility is that the increases in *Hsp *gene expression are secondary to increases in the expression and activity of HSF1. However, we did not observe changes in *Hsf1 *expression in our transcript profiling studies and earlier studies showed that HSF1 and HSF2 expression and binding to HSE was not altered by WY exposure in the rat liver [64]. To help determine whether regulation of *Hsp *gene expression is direct or indirect, we examined their promoters and found that only a few genes possess a putative PPRE(s) (data not shown) indicating indirect activation.

Many *Hsp *genes may be regulated indirectly through increases in oxidative stress. There is abundant evidence for the increased expression of chaperone gene expression under conditions that also increase oxidative stress [5,45]. PPC exposure leads to increases in oxidative stress and lipid peroxidation mediated through increased activities of enzymes that generate reactive oxygen species [10]. Although direct evidence that Hsp genes are activated by PPC-mediated increases in oxidative stress is lacking, the absolute increases in expression of some *Hsp *genes was higher in mice nullizygous for Nrf2, a transcription factor activated by oxidative stress that regulates genes that decrease oxidative stress. Thus, in the absence of Nrf2, increased levels of oxidative stress may have contributed to the greater increases in the *Hsp *genes by WY [12]. However, we cannot rule out the induction of a HSP1 cofactor secondary to PPARα induction." In summary, PPARα may regulate the *Hsp *genes in a species-specific manner secondarily to increases in oxidative stress. Further work is needed to confirm this hypothesis.

## Conclusions

Our microarray analysis of HS in the livers of mice uncovered a dramatic influence of PPARα on the overall gene expression pattern. There were remarkable differences in the transcriptional response to HS between wild-type and PPARα-null mice. A number of HS genes required PPARα for induction. Many mitochondrial genes were uniquely down-regulated in PPARα-null but not wild-type mice. Pretreatment of PPARα-null mice with WY prevented the down-regulation of these genes possibly through the increased expression of PGC-1β and regulated genes. Our findings support the hypothesis that PPARα in the liver acts as an integrator of chemical and physical stress signals manifested in the regulation of stress gene responses in the liver.

## Abbreviations

DEHP: di-(2-ethylhexyl)phthalate; HSE: heat shock elements; HSF: heat shock factor; HSP: heat shock proteins; PCR: polymerase chain reaction; PPAR: peroxisome proliferator-activated receptor; PPC: peroxisome proliferator chemical; PPRE: peroxisome proliferator response element; WY: WY-14,643.

## Competing interests

The authors declare that they have no competing interests.

## Authors' contributions

BV analyzed the microarray and clinical chemistry data and helped to draft the manuscript. SA generated and analyzed the microarray data and analyzed the RT-PCR data. HBB analyzed the western data. HR analyzed the microarray data. SK generated the microarray data. SJ and RS analyzed the MEF microarray dataset. JCC conceived of the study, participated in study design and animal studies, generated western data, analyzed microarray data and helped to draft the manuscript. All authors read and approved the final manuscript.

## Supplementary Material

Additional file 1 **Sequences of primers**. Sequences of primers used in TaqMan studies. Click here for file

Additional file 2 **Gene expression changes after WY, heat shock or heat shock+WY in wild-type and PPARα-null mouse livers**. Table represents the gene annotations and fold changes for gene expression changes after WY, heat shock or heat shock+WY in wild-type and PPARα-null mouse livers. Click here for file

Additional file 3 **Table of genesets significantly up-regulated by heat shock in wild-type mice**. Table describes GSEA genesets significantly up-regulated by heat shock in wild-type mice. Click here for file

Additional file 4 **Table of genesets significantly up-regulated by heat shock in PPARα-null mice**. Table describes GSEA genesets significantly up-regulated by heat shock in PPARα-null mice. Click here for file

Additional file 5 **Table of genesets significantly down-regulated by heat shock in PPARα-null mice**. Table describes GSEA genesets significantly down-regulated by heat shock in PPARα-null mice. Click here for file

Additional file 6 **Table of transcription factor genesets significantly up-regulated by heat shock in wild-type mice**. Table describes the GSEA transcription factor genesets significantly up-regulated by heat shock in wild-type mice. Click here for file

Additional file 7 **Table of transcription factor genesets significantly up-regulated by heat shock in PPARα-null mice**. Table describes the GSEA transcription factor genesets significantly up-regulated by heat shock in PPARα-null mice. Click here for file

Additional file 8 **Analysis of interactions between WY and HS in wild-type mice**. Interactions between WY and HS in wild-type mice using Ingenuity Pathway Analysis Tool. Click here for file

Additional file 9 **Canonical pathways analysis using Ingenuity**. Canonical pathways in an Ingenuity comparison between genes only regulated by WY or only by WY+HS in wild-type mice. Click here for file

Additional file 10 **Excel spreadsheet of genes regulated by heat shock or by strain differences between wild-type and HSF1-null mice**. Genes were originally from the Trinklein et al. (2004) study. Excel spreadsheet of genes regulated by heat shock or by strain differences between wild-type and HSF1-null mice. Click here for file

Additional file 11 **Figures of expression of heat shock genes in wild-type and HSF1-null mouse embryonic fibroblasts**. Mouse embryonic fibroblasts were given a HS and cells were harvested at the indicated times as described (Trinklein et al., 2004). Genes which exhibited significant changes after HS or that exhibited significant differences between wild-type and HSF1-null strains were identified as described in the Methods. A. HSF1-dependent HS genes. B. HSF1-independent HS genes. C. Genes which exhibited differences in expression between control wild-type and control HSF1-null strains. Click here for file
