# Supplementary material for: Analysis of the heat shock response in mouse liver reveals transcriptional dependence on the nuclear receptor peroxisome proliferator-activated receptor α (PPARα)
Source: BMC Genomics. 2010 Jan 7;11:16. doi: 10.1186/1471-2164-11-16 (PMC2823686; doi:10.1186/1471-2164-11-16)
Supplement: Additional file 1 — Sequences of primers. Sequences of primers used in TaqMan studies. [file 1471-2164-11-16-S1.DOC]

**Vallanat et al. Additional File 1**

**Additional File 1 Sequences of primers**

| Gene | Accession Number | Forward | Probe | Reverse |
| --- | --- | --- | --- | --- |
| *Hsp90aa1* | J04633 | TTCACGAGGACTCTCAGAATCG | AGAAGCTTTCAGAGCTGTTGCGG | GTCCCCAGAAGCAGATGTGTAGT |
| *Hsph1* | L40406 | TCCGGCATTTAAAGTTAGAGAGTTC | TGTCACCGATGCAGTTCCTTTT | CGTGGTTCCAGACCAGAGATATT |
| *Dnaja1* | AF055664 | TCAGGTAGAACTGGTGGACTTTGA | CCAAATCAGGAAAGACGGCGTCA | TCATCCTCATACGCTTCTCCATT |
| *Stip1* | U27830 | TCAGACCTGGGCACGAAACT | CAGGATCCCCGGGTGATGACT | CCCCAAGGAGGACACTCAGA |
| *Tubb2* | M28739 | CGCATCTCGGAGCAGTTCA | TGCCATGTTCCGGCGCA | CGTGTACCAGTGCAGGAAAGC |
| *Uchl1* | AB025313 | ACCAAGACAAGCTGGAATTTGAG | ATGGATCCGTCCTGAAACAGTTTC | GGACAGCTTCTCCGTTTCAGA |

**Sequences of primers used in TaqMan studies.**
