# Supplementary material for: Analysis of the heat shock response in mouse liver reveals transcriptional dependence on the nuclear receptor peroxisome proliferator-activated receptor α (PPARα)
Source: BMC Genomics. 2010 Jan 7;11:16. doi: 10.1186/1471-2164-11-16 (PMC2823686; doi:10.1186/1471-2164-11-16)
Supplement: Additional file 3 — Table of genesets significantly up-regulated by heat shock in wild-type mice. Table describes GSEA genesets significantly up-regulated by heat shock in wild-type mice. [file 1471-2164-11-16-S3.DOC]

**Additional File 3. Genesets significantly up-regulated by heat shock in wild-type mice.**

| **NAME** | **SIZE** | **NES** | **NOM p-val** | **FDR q-val** | **FWER p-val** |
| --- | --- | --- | --- | --- | --- |
| HSA03010_RIBOSOME | 44 | -2.73853 | 0 | 0 | 0 |
| CANCER_NEOPLASTIC_META_UP | 53 | -2.70706 | 0 | 0 | 0 |
| PENG_RAPAMYCIN_DN | 156 | -2.62464 | 0 | 0 | 0 |
| ZELLER_MYC_UP | 23 | -2.60835 | 0 | 0 | 0 |
| HSA03050_PROTEASOME | 22 | -2.5169 | 0 | 0 | 0 |
| PROTEASOME_DEGRADATION | 29 | -2.51664 | 0 | 0 | 0 |
| PENG_LEUCINE_DN | 118 | -2.50775 | 0 | 1.94E-04 | 0.001 |
| MYC_TARGETS | 38 | -2.4772 | 0 | 1.70E-04 | 0.001 |
| PROTEASOMEPATHWAY | 21 | -2.41031 | 0 | 1.51E-04 | 0.001 |
| UVB_NHEK2_UP | 51 | -2.40735 | 0 | 1.36E-04 | 0.001 |
| PENG_GLUTAMINE_DN | 194 | -2.37116 | 0 | 3.93E-04 | 0.003 |
| PROTEASOME | 17 | -2.34176 | 0 | 4.81E-04 | 0.004 |
| RIBOSOMAL_PROTEINS | 55 | -2.29704 | 0 | 7.93E-04 | 0.007 |
| HSA00100_BIOSYNTHESIS_OF_STEROIDS | 22 | -2.26671 | 0 | 0.001146 | 0.011 |
| TARTE_PLASMA_BLASTIC | 244 | -2.20082 | 0 | 0.002792 | 0.029 |
| LIZUKA_G1_SM_G2 | 17 | -2.18744 | 0 | 0.003057 | 0.034 |
| FLECHNER_KIDNEY_TRANSPLANT_WELL_UP | 380 | -2.185 | 0 | 0.002877 | 0.034 |
| CPR_NULL_LIVER_UP | 25 | -2.16414 | 0 | 0.003431 | 0.042 |
| BLEO_MOUSE_LYMPH_HIGH_24HRS_DN | 29 | -2.15797 | 0 | 0.003479 | 0.045 |
| TRNA_SYNTHETASES | 15 | -2.1477 | 0 | 0.003948 | 0.053 |

Size indicates the number of genes which overlap between the gene set and those genes on the U74Av2 chip. NES, enrichment score normalised for differences in gene set size; NOM, nominal. p-values indicated as 0 are < 0.001. Please see the GSEA User Guide or Subramanian et al. (2005) for further definitions and algorithm details.
