# Supplementary material for: Analysis of the heat shock response in mouse liver reveals transcriptional dependence on the nuclear receptor peroxisome proliferator-activated receptor α (PPARα)
Source: BMC Genomics. 2010 Jan 7;11:16. doi: 10.1186/1471-2164-11-16 (PMC2823686; doi:10.1186/1471-2164-11-16)
Supplement: Additional file 4 — Table of genesets significantly up-regulated by heat shock in PPARα-null mice. Table describes GSEA genesets significantly up-regulated by heat shock in PPARα-null mice. [file 1471-2164-11-16-S4.DOC]

**Additional File 4. Genesets significantly up-regulated by heat shock in PPARα**-null mice.

| **NAME** | **SIZE** | **NES** | **NOM p-val** | **FDR q-val** | **FWER p-val** |
| --- | --- | --- | --- | --- | --- |
| LEE_DENA_UP | 43 | -2.664 | 0 | 0 | 0 |
| IGLESIAS_E2FMINUS_UP | 137 | -2.55093 | 0 | 0 | 0 |
| LEE_MYC_E2F1_UP | 42 | -2.53255 | 0 | 0 | 0 |
| LEE_E2F1_UP | 46 | -2.46419 | 0 | 0 | 0 |
| LEE_MYC_TGFA_UP | 49 | -2.33884 | 0 | 0 | 0 |
| CARIES_PULP_UP | 150 | -2.27506 | 0 | 2.24E-04 | 0.001 |
| LEE_ACOX1_UP | 47 | -2.25508 | 0 | 1.92E-04 | 0.001 |
| CARIES_PULP_HIGH_UP | 62 | -2.2049 | 0 | 3.35E-04 | 0.002 |
| LEE_CIP_UP | 49 | -2.18278 | 0 | 2.98E-04 | 0.002 |
| ROS_MOUSE_AORTA_DN | 67 | -2.18266 | 0 | 2.68E-04 | 0.002 |
| ICHIBA_GVHD | 335 | -2.18008 | 0 | 3.58E-04 | 0.003 |
| NI2_MOUSE_UP | 37 | -2.14012 | 0 | 8.77E-04 | 0.008 |
| IGF_VS_PDGF_UP | 43 | -2.13109 | 0 | 9.15E-04 | 0.009 |
| IRITANI_ADPROX_VASC | 116 | -2.08381 | 0 | 0.00152078 | 0.016 |
| STRIATED_MUSCLE_CONTRACTION | 30 | -2.0778 | 0 | 0.00159483 | 0.018 |
| BASSO_GERMINAL_CENTER_CD40_UP | 72 | -2.05408 | 0 | 0.00248231 | 0.03 |
| ROSS_CBF_MYH | 34 | -2.05393 | 0.002288 | 0.00233629 | 0.03 |
| AGEING_KIDNEY_UP | 217 | -2.0213 | 0 | 0.00317442 | 0.043 |

Size indicates the number of genes which overlap between the gene set and those genes on the U74Av2 chip. NES, enrichment score normalised for differences in gene set size; NOM, nominal. p-values indicated as 0 are < 0.001. Please see the GSEA User Guide or Subramanian et al. (2005) for further definitions and algorithm details.
