# Supplementary material for: Analysis of the heat shock response in mouse liver reveals transcriptional dependence on the nuclear receptor peroxisome proliferator-activated receptor α (PPARα)
Source: BMC Genomics. 2010 Jan 7;11:16. doi: 10.1186/1471-2164-11-16 (PMC2823686; doi:10.1186/1471-2164-11-16)
Supplement: Additional file 5 — Table of genesets significantly down-regulated by heat shock in PPARα-null mice. Table describes GSEA genesets significantly down-regulated by heat shock in PPARα-null mice. [file 1471-2164-11-16-S5.DOC]

**Additional File 5. Genesets significantly down-regulated by heat shock in PPARα-null mice.**

| **NAME** | **SIZE** | **NES** | **NOM p-val** | **FDR q-val** | **FWER p-val** |
| --- | --- | --- | --- | --- | --- |
| MOOTHA_VOXPHOS | 60 | 2.004829 | 0 | 0.00228645 | 0.054 |
| HSA00650_BUTANOATE_METABOLISM | 28 | 2.014883 | 0 | 0.00190032 | 0.043 |
| LEE_MYC_TGFA_DN | 49 | 2.044003 | 0 | 0.00108338 | 0.023 |
| LEE_CIP_DN | 48 | 2.078785 | 0 | 4.51E-04 | 0.009 |
| VALINE_LEUCINE_AND_ISOLEUCINE_DEGRADATION | 26 | 2.090548 | 0 | 4.31E-04 | 0.008 |
| HSA00071_FATTY_ACID_METABOLISM | 29 | 2.0988 | 0 | 4.03E-04 | 0.007 |
| LIZUKA_G2_GR_G3 | 20 | 2.099121 | 0 | 4.36E-04 | 0.007 |
| LEE_MYC_E2F1_DN | 47 | 2.11333 | 0 | 4.76E-04 | 0.007 |
| PGC | 310 | 2.123017 | 0 | 4.50E-04 | 0.006 |
| FLECHNER_KIDNEY_TRANSPLANT_REJECTION_DN | 367 | 2.133886 | 0 | 2.51E-04 | 0.003 |
| LEE_DENA_DN | 57 | 2.177487 | 0 | 0 | 0 |
| HSA00280_VALINE_LEUCINE_AND_ISOLEUCINE_DEGRADATION | 33 | 2.211185 | 0 | 0 | 0 |
| HSA00380_TRYPTOPHAN_METABOLISM | 34 | 2.238811 | 0 | 0 | 0 |
| HUMAN_MITODB_6_2002 | 291 | 2.258257 | 0 | 0 | 0 |
| HSIAO_LIVER_SPECIFIC_GENES | 173 | 2.267491 | 0 | 0 | 0 |
| MITOCHONDRIA | 293 | 2.282037 | 0 | 0 | 0 |
| IDX_TSA_UP_CLUSTER5 | 90 | 2.285024 | 0 | 0 | 0 |
| HCC_SURVIVAL_GOOD_VS_POOR_UP | 81 | 2.342778 | 0 | 0 | 0 |

Size indicates the number of genes which overlap between the gene set and those genes on the U74Av2 chip. NES, enrichment score normalised for differences in gene set size; NOM, nominal. p-values indicated as 0 are < 0.001. Please see the GSEA User Guide or Subramanian et al. (2005) for further definitions and algorithm details.
