# Supplementary material for: Analysis of the heat shock response in mouse liver reveals transcriptional dependence on the nuclear receptor peroxisome proliferator-activated receptor α (PPARα)
Source: BMC Genomics. 2010 Jan 7;11:16. doi: 10.1186/1471-2164-11-16 (PMC2823686; doi:10.1186/1471-2164-11-16)
Supplement: Additional file 6 — Table of transcription factor genesets significantly up-regulated by heat shock in wild-type mice. Table describes the GSEA transcription factor genesets significantly up-regulated by heat shock in wild-type mice. [file 1471-2164-11-16-S6.DOC]

Additional File 6. Transcription factor genesets significantly up-regulated by heat shock in wild-type mice.

| NAME | DESCRIPTION | SIZE | NES | NOM p-val | FDR q-val | FWER p-val |
| --- | --- | --- | --- | --- | --- | --- |
| TTCNRGNNNNTTC_V$HSF_Q6 | Heat shock factor | 83 | -1.98958 | 0 | 0.018106 | 0.018 |
| GGCNKCCATNK_UNKNOWN | Unknown | 55 | -1.86331 | 0 | 0.032051 | 0.063 |
| GCGNNANTTCC_UNKNOWN | Unknown | 59 | -1.8318 | 0 | 0.028539 | 0.083 |
| KCCGNSWTTT_UNKNOWN | Unknown | 64 | -1.78705 | 0 | 0.031819 | 0.118 |
| CGGAARNGGCNG_UNKNOWN | Unknown | 24 | -1.74989 | 0.006969 | 0.038404 | 0.175 |
| V$HTF_01^1^ | X-box binding protein 1;  hepatocarcinogenesis transcription factor | 32 | -1.50019 | 0.02847 | 0.136382 | 0.811 |
| RCGCANGCGY_V$NRF1_Q6^1^ | Nuclear respiratory factor 1 | 411 | -1.46889 | 0 | 0.160392 | 0.878 |
| V$HSF_Q6^1^ | Heat shock factor | 101 | -1.40271 | 0.015385 | 0.229232 | 0.963 |
| V$XBP1_01^1^ | X-box binding protein 1 | 70 | -1.33716 | 0.046512 | 0.280813 | 0.994 |

Size indicates the number of genes which overlap between the gene set and those genes on the U74Av2 chip. NES, enrichment score normalised for differences in gene set size; NOM, nominal. p-values indicated as 0 are < 0.001. Please see the GSEA User Guide or Subramanian et al. (2005) for further definitions and algorithm details.

^1^Indicates gene sets that did not reach statistical significance but may have biological significance (discussed in text).
