# Supplementary material for: Analysis of the heat shock response in mouse liver reveals transcriptional dependence on the nuclear receptor peroxisome proliferator-activated receptor α (PPARα)
Source: BMC Genomics. 2010 Jan 7;11:16. doi: 10.1186/1471-2164-11-16 (PMC2823686; doi:10.1186/1471-2164-11-16)
Supplement: Additional file 7 — Table of transcription factor genesets significantly up-regulated by heat shock in PPARα-null mice. Table describes the GSEA transcription factor genesets significantly up-regulated by heat shock in PPARα-null mice. [file 1471-2164-11-16-S7.DOC]

**Additional File 7. Transcription factor genesets significantly up-regulated by heat shock in PPARα**-null mice.

| NAME | DESCRIPTION | SIZE | NES | NOM p-val | FDR q-val | FWER p-val |
| --- | --- | --- | --- | --- | --- | --- |
| CCAWWNAAGG_V$SRF_Q4 | Serum response factor | 44 | -1.86394 | 0.002198 | 0.05906 | 0.035 |
| GGARNTKYCCA_UNKNOWN | Unknown | 43 | -1.85152 | 0 | 0.038764 | 0.045 |

Size indicates the number of genes which overlap between the gene set and those genes on the U74Av2 chip. NES, enrichment score normalised for differences in gene set size; NOM, nominal. p-values indicated as 0 are < 0.001. Please see the GSEA User Guide or Subramanian et al. (2005) for further definitions and algorithm details.
