# Supplementary material for: Analysis of the heat shock response in mouse liver reveals transcriptional dependence on the nuclear receptor peroxisome proliferator-activated receptor α (PPARα)
Source: BMC Genomics. 2010 Jan 7;11:16. doi: 10.1186/1471-2164-11-16 (PMC2823686; doi:10.1186/1471-2164-11-16)
Supplement: Additional file 8 — Analysis of interactions between WY and HS in wild-type mice. Interactions between WY and HS in wild-type mice using Ingenuity Pathway Analysis Tool. [file 1471-2164-11-16-S8.DOC]

**Additional File 8. Analysis of interactions between WY and HS in wild-type mice.**

To determine whether there were interactions between WY and HS in wild-type mice, we compared the genes regulated by WY or by WY+HS co-treatment. Most of the genes (1117 genes) were similarly expressed under both conditions. However, there were 597 or 301 genes that were uniquely regulated by WY or by WY+HS, respectively. Functional analysis of these genes by Ingenuity revealed an overrepresentation of a number of canonical pathways including biosynthesis of steroids, mitochondrial dysfunction, oxidative phosphorylation, ubiquinone biosynthesis and VEGF signaling (Additional File 11). Canonical pathways that were overrepresented in the WY+HS group included protein ubiquination pathway, tyrosine metabolism, glycolysis/gluconeogenesis, glycine, serine and threonine metabolism and purine metabolism.

HS prevented WY from uniformly up-regulating genes involved in cholesterol synthesis including *Cyp8b1, Dhcr7, Ebp, Fdft1, Fdps, Idi1, Lss, Sc5dl,* and *Sqle*. HS also prevented WY from activating ~20 genes linked to mitochondrial dysfunction and oxidative phosphorylation. Finally, despite the ability of HSF1 to protect mice from an acute exposure to LPS (Xiao et al., 1999), HS reversed the almost uniform down-regulation of genes that are part of the canonical pathways Il-4, Il-15 and Il-17 signaling (Additional File 11). Overall, these results indicate that there were interactions between WY and HS in wild-type mice distinct from those in PPARα-null mice. Further studies are needed to determine the functional consequences and molecular basis for these interactions.

Xiao X, Zuo X, Davis AA, McMillan DR, Curry BB, Richardson JA, Benjamin IJ: [**HSF1 is required for extra-embryonic development, postnatal growth and protection during inflammatory responses in mice.**](http://www.ncbi.nlm.nih.gov/pubmed/10545106?ordinalpos=2&itool=EntrezSystem2.PEntrez.Pubmed.Pubmed_ResultsPanel.Pubmed_DefaultReportPanel.Pubmed_RVDocSum) *EMBO J.* 1999 **18**(21):5943-52.
