# Supplementary material for: Analysis of the heat shock response in mouse liver reveals transcriptional dependence on the nuclear receptor peroxisome proliferator-activated receptor α (PPARα)
Source: BMC Genomics. 2010 Jan 7;11:16. doi: 10.1186/1471-2164-11-16 (PMC2823686; doi:10.1186/1471-2164-11-16)
Supplement: Additional file 11 — Figures of expression of heat shock genes in wild-type and HSF1-null mouse embryonic fibroblasts. Mouse embryonic fibroblasts were given a HS and cells were harvested at the indicated times as described (Trinklein et al., 2004). Genes which exhibited significant changes after HS or that exhibited significant differences between wild-type and HSF1-null strains were identified as described in the Methods. A. HSF1-dependent HS genes. B. HSF1-independent HS genes. C. Genes which exhibited differences in expression between control wild-type and control HSF1-null strains. [file 1471-2164-11-16-S11.PPT]

## Slide 1
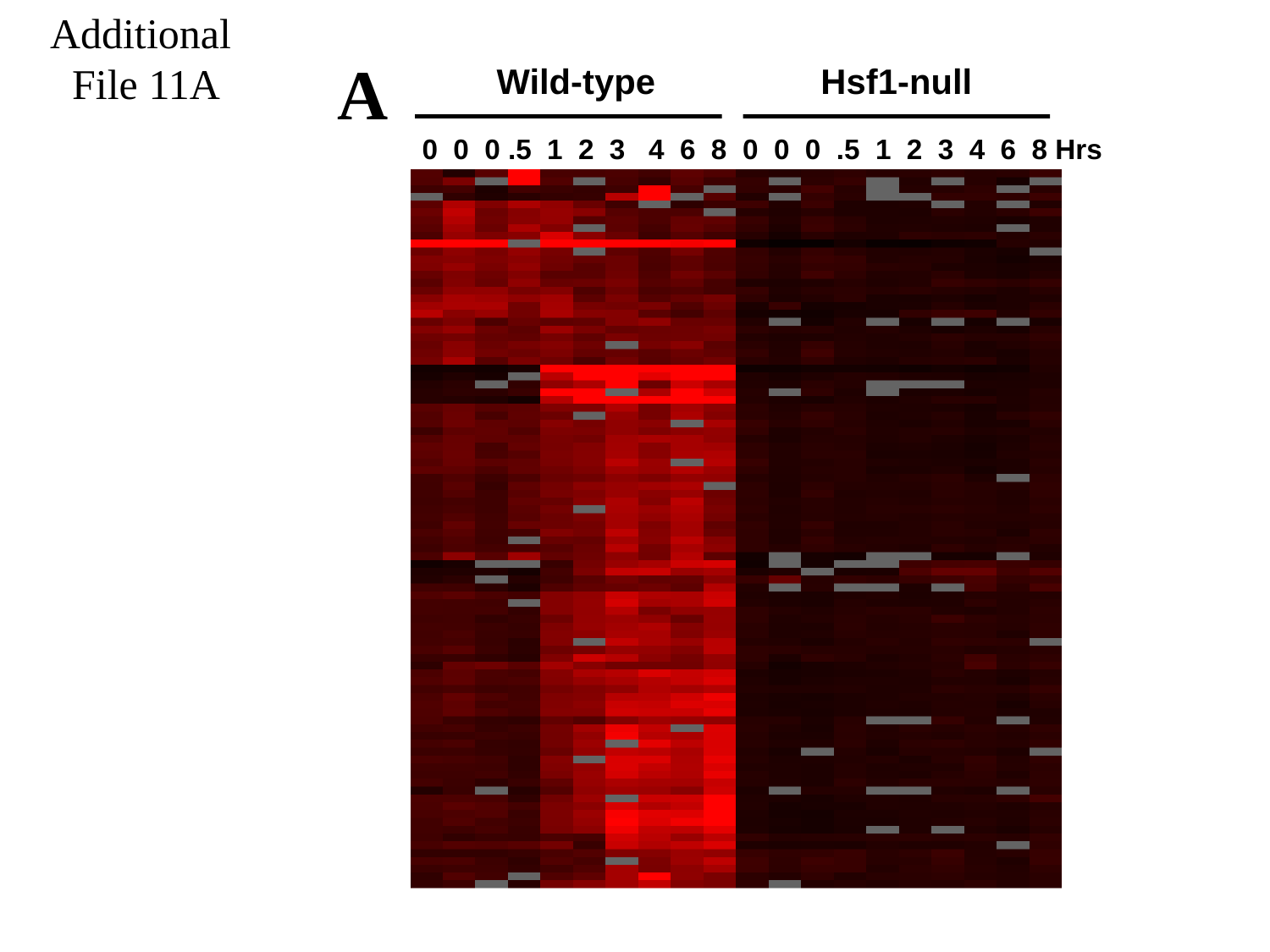

Additional
File 11A
A
 Wild-type Hsf1-null
 0 0 0 .5 1 2 3 4 6 8 0 0 0 .5 1 2 3 4 6 8 Hrs

## Slide 2
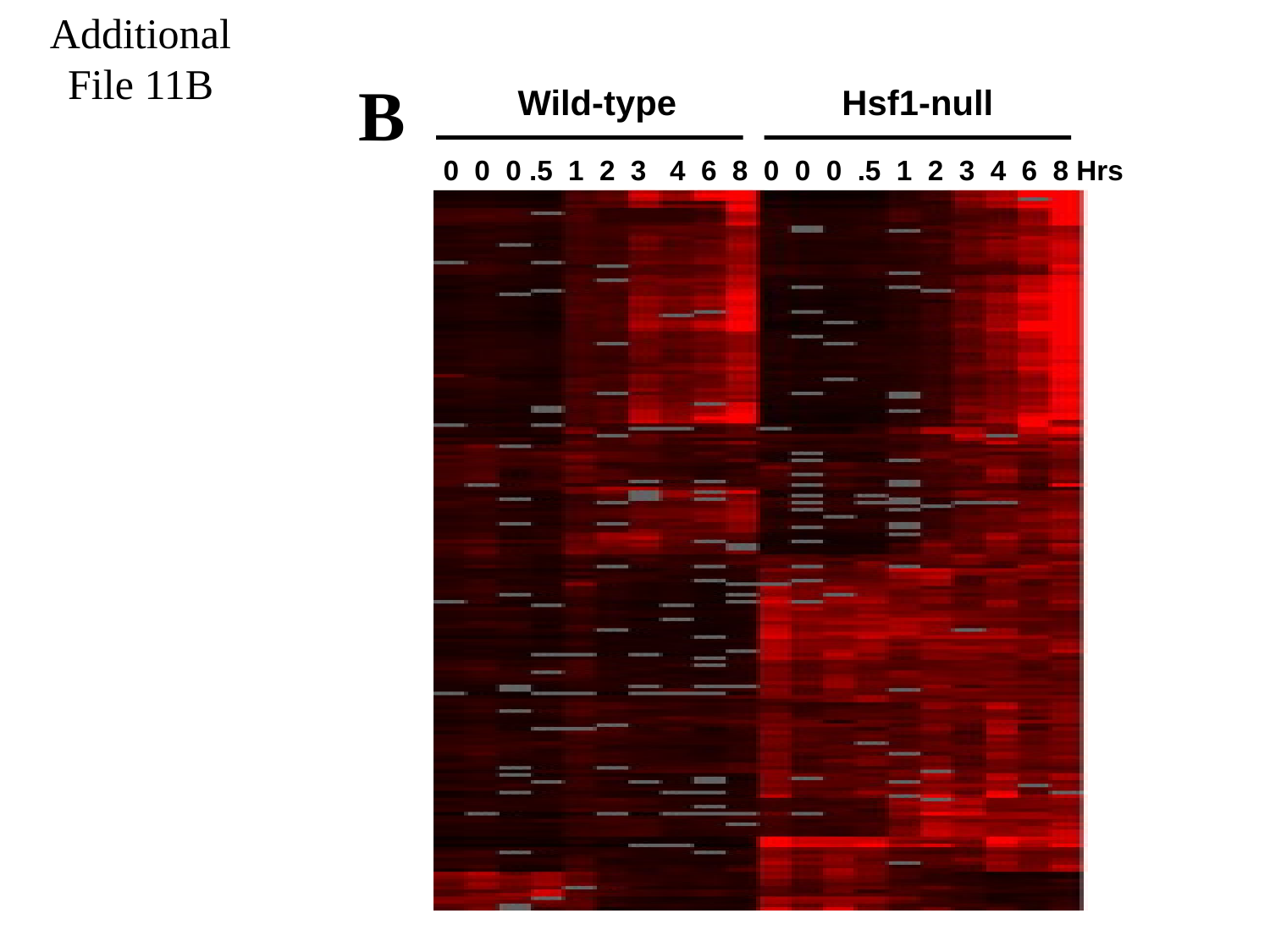

Additional
 File 11B
B
 Wild-type Hsf1-null
 0 0 0 .5 1 2 3 4 6 8 0 0 0 .5 1 2 3 4 6 8 Hrs

## Slide 3
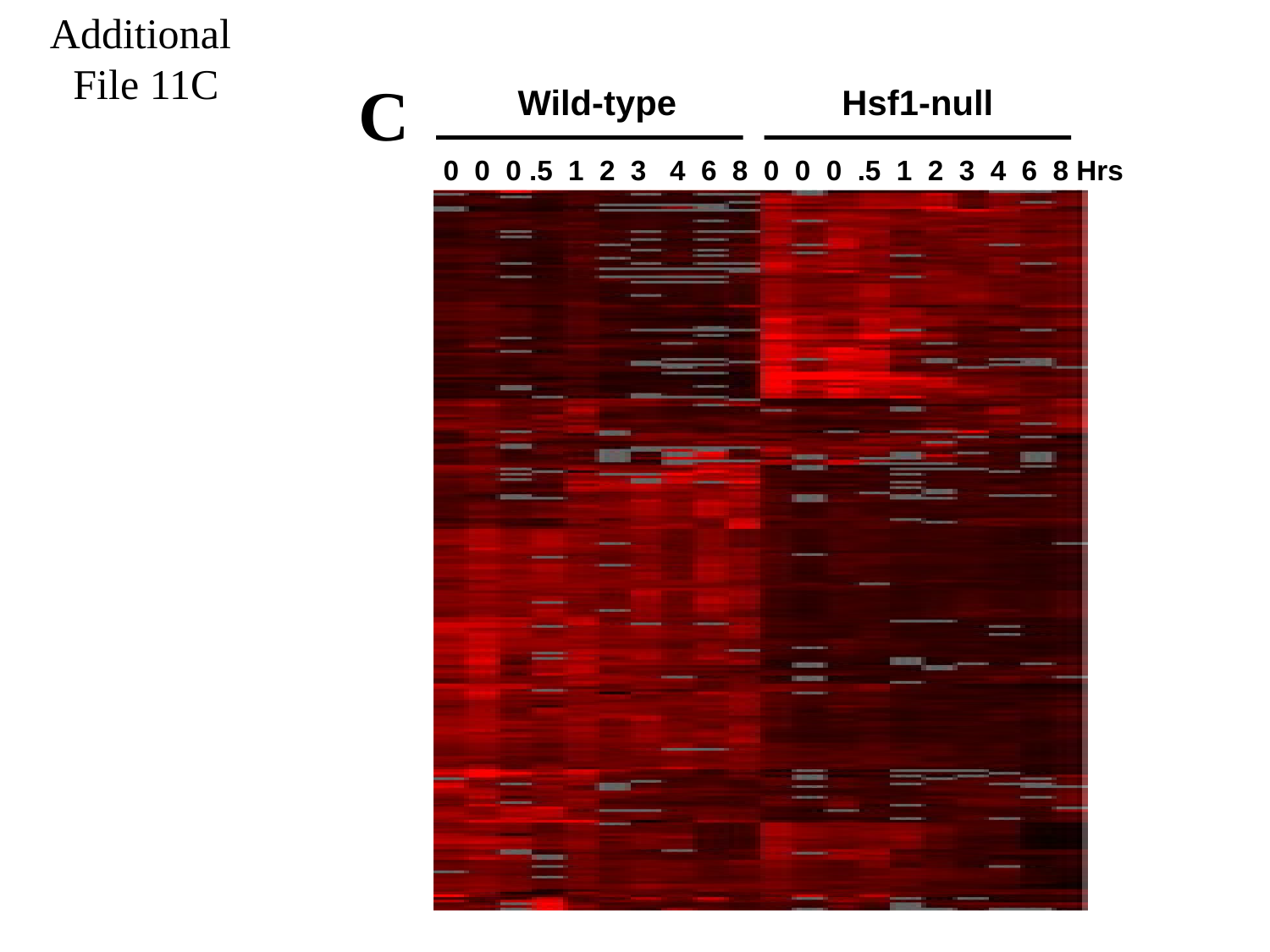

Additional
File 11C
C
 Wild-type Hsf1-null
 0 0 0 .5 1 2 3 4 6 8 0 0 0 .5 1 2 3 4 6 8 Hrs
